# Intra‐ and Inter‐Specific Ecological Impacts Vary Across a Gradient of Abundance of an Invasive Species, *Bothriochloa ischaemum*, in a Mixed‐Grass Prairie

**DOI:** 10.1002/ece3.73212

**Published:** 2026-03-10

**Authors:** Joshua D. Kouri, Emma Rust, Lara Souza

**Affiliations:** ^1^ School of Biological Sciences University of Oklahoma Norman Oklahoma USA; ^2^ Coastal San Luis Resource Conservation District Morro Bay California USA; ^3^ Oklahoma Biological Survey University of Oklahoma Norman Oklahoma USA

**Keywords:** *Bothriochloa ischaemum*, functional traits, impact, invasion gradient, Oklahoma, *Schizachyrium scoparium*

## Abstract

Managing biological invasions is one of the top priorities of biodiversity conservation. Invasive plants are a well‐known threat to native plant and animal communities, and understanding their ecological impacts is critical to developing individualized management strategies. While much is known about the impacts of invasive plants, there are still questions about the per capita effects along invasion abundance gradients across levels of biological organization. In this study we investigate how the ecological impacts of the invasive grass *Bothriochloa ischaemum* vary across a gradient of invasion and whether effects are consistent across population (abundance and functional traits of a dominant native grass, *Schizachyrium scoparium* ) and community (species richness and composition) levels. We found that most of the ecological impacts of *B. ischaemum* scale linearly with its abundance across population and community levels. Increasing invasion reduces the height and abundance of the dominant native *S. scoparium* individuals and shifts their functional trait composition. Increasing invasion also reduces cover of native C_3_ and C_4_ grasses, total foliar cover, subdominant foliar cover, species richness, and leads to shifts in species and functional group composition. However, the impact on legume abundance saturated at low invader abundance (1%–15% cover) and remained constant as invader abundance increased. We show that the direct ecological impacts of invasive species may be compounded by shifts in the functional traits of dominant native species toward more conservative traits and shifts in species and functional group composition, leading toward a shift in population and community structure and function.

## Graphical abstract

The invasive grass *Bothriochloa ischaemum* negatively impacts native plant communities along an invasion gradient, reducing native grass abundance, plant species richness, and altering functional traits and overall community structure. Most intra‐ and inter‐specific effects increase with greater invader abundance.

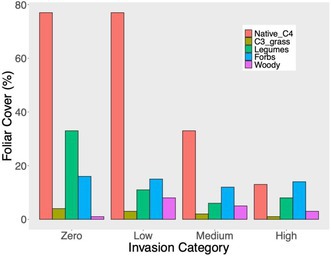

## 1 Introduction

Density‐dependent, per‐capita invasive plant impacts are key to understanding and predicting biological invasions, especially across levels of ecological organization (Dietzsch et al. 2011; Funk et al. 2016; Sodhi et al. 2019; Damasceno and Fidelis 2023). There is a need for the quantitative assessment of ecological impacts across a gradient of invader abundance levels (Ehrenfeld 2010; Vilà et al. 2011; Hulme et al. 2013; Scasta et al. 2015; Damasceno and Fidelis 2023). Incorporating these gradients into invasion studies can provide more clarity on the relationship between impacts and abundance than comparisons between uninvaded and highly invaded sites (Hulme et al. 2013; Damasceno and Fidelis 2023). It is important to understand if impacts scale linearly with abundance or if there are thresholds below which invaders have little impact or above which impacts begin to saturate (Vilà et al. 2011). Furthermore, employing a gradient design allows for the utilization of more powerful regression analysis (rather than ANOVA) and offers a quantitative description of the relationship between invader abundance and impact (Cottingham et al. 2005). Adding an invasion gradient lens to functional trait studies allows a unique perspective into the consequences of invaders on species‐level characteristics within a larger community (Vilà et al. 2011). It can identify to what degree an invasive species must expand to shift dominant, and possibly secondary, native species' functional traits via expression of phenotypic responses (Sodhi et al. 2019; Damasceno and Fidelis 2023). This study contributes to filling both of the above knowledge gaps by incorporating population‐level functional trait and community level analyses into an investigation of impacts across a gradient of invader abundance across two levels of organization.

Functional traits are the measurable attributes of a plant that can both respond to *and* influence its interactions with its biotic and abiotic environment (Drenovsky et al. 2012; Pérez‐Harguindeguy et al. 2013). Plant functional traits can have significant effects on the surrounding community and its resulting ecosystem services and functioning (McGill et al. 2006; de Bello et al. 2010). The traits of the dominant–i.e. common and abundant−plant species tend to exert the greatest influence on ecosystem processes (Grime 1998; Díaz and Cabido 2001; Mokany et al. 2008; Roscher et al. 2012; Finegan et al. 2015; Hou et al. 2023). The important role of functional traits leads to two pathways for invasive plants to impact communities and ecosystems. First, when invaders dominate native communities, differences between native and invasive functional traits can directly alter ecosystem functioning (Daehler 2003; Funk and Vitousek 2007; Ehrenfeld 2010; Drenovsky et al. 2012). However, if the presence of an invader can trigger changes in the functional trait expression of the dominant native species as well, that could provide a second pathway to alter ecosystem functioning. Although it is important to acknowledge the inherent variation in native species functional traits prior to invasive species entering an ecosystem (Crawford et al. 2019; Ren et al. 2024), plant species are known to exhibit high levels of phenotypic plasticity in response to their surroundings, and invasive plants are known to alter environmental conditions (Vilà et al. 2011), possibly triggering a phenotypic response from native species and entire food webs. Ecological impacts arising directly from the functional traits of invasive plants could be compounded by changes to the traits of the dominant native plants that scale up to indirectly impact communities and ecosystems.

The prairie ecosystems of the central United States provide an excellent opportunity for studying the impacts of invasive plants, given the well‐documented introductions and establishment of a variety of non‐native species (D'Antonio and Vitousek 1992; Williams and Baruch 2000). *Bothriochloa ischaemum*, a C_4_ grass originating in Eurasia, is one such species that has become a problematic invader in the Great Plains region of the United States (Gabbard and Fowler 2007). *Bothriochloa ischaemum* has been intentionally seeded on both private lands and along public roadsides through several state and federal agencies for use as cattle fodder and erosion control (Gabbard and Fowler 2007; Wilson et al. 2012). *B. ischaemum* has been shown to reduce the abundance and diversity of native plant communities (Hickman et al. 2006; Gabbard and Fowler 2007; Robertson and Hickman 2012), inhibit the growth of native plants (Schmidt et al. 2008), reduce arthropod abundance and bird diversity (Hickman et al. 2006), and replace the dominant native C4 grasses in the habitat (Robertson and Hickman 2012). The ecosystem impacts of *B. ischaemum* invasion are poorly known, but it has been shown to directly reduce plant‐available soil nitrogen (Basham 2013) and may indirectly alter nutrient cycling and pollination services through its impacts on insect communities (Litt and Steidl 2010). The wide breadth of negative impacts caused by *B. ischaemum* invasion makes this grass one of the most detrimental exotic forage species (Scasta et al. 2015). The goal of this study is to investigate how the invasive grass *Bothriochloa ischaemum* impacts the native plant community across a gradient of invasion, considering both within‐species impacts (on the functional traits of the native dominant grass, *Schizachyrium scoparium* ) and across‐species impacts (on species and functional group composition). We specifically asked (1) Is there a negative relationship between the abundance of the invader and the abundance of the dominant native grass? (2) Do changes in the abundance of the invasive grass lead to changes in the mean, variance, or composition of functional trait values for the dominant native grass? (3) Does increasing abundance of the invasive grass change the richness or composition of the native plant community? We hypothesized that as the invasive species increased in abundance, the native dominant grass would decrease in abundance, mean and variance of trait values and shift its functional trait composition toward conservative traits, compounding changes in the community and ecosystem.

## 2 Methods

### 2.1 Study Site

We conducted our study in a temperate mixed‐grass prairie at the Kessler Atmospheric and Ecological Field Station (KAEFS, http://kaefs.ou.edu/), located in central Oklahoma, USA (34°59′ N, 97°31′ W). The area was farmed until 1973 and has since been subject to light grazing in designated areas (Xu et al. 2013). We conducted our study in two sections of KAEFS that have not been farmed or grazed for at least 40 years prior to the start of the experiment (Shi et al. 2015; Castillioni et al. 2020) and are dominated by warm‐season C_4_ bunch grasses with a diverse mixture of subdominant forbs (Shi et al. 2015). Mean annual precipitation was 885 mm for the period of 1994–2018 and the mean annual temperature was 16.2°C from 1997 to 2018 (Oklahoma Climatological Survey, Norman, OK, USA). The monthly mean temperature ranges from 4.4°C in January to 27.7°C in July (Oklahoma Climatological Survey, Norman, OK, USA). The soil at KAEFS is a Nash‐Lucien complex with neutral pH, a high water holding capacity (approximately 37%), and a moderately penetrable root zone, and extends to a depth of about 70 cm (Xu et al. 2013).

### 2.2 Study Design

We located study plots across two locations (sub‐sites) consisting of mixed‐grass prairie surrounded by encroaching eastern red cedar ( *Juniperus virginiana* ) woodlands. These sites are dominated by native C_4_ grasses such as little bluestem ( *Schizachyrium scoparium* ), Indian grass ( *Sorghastrum nutans* ), and tall dropseed ( *Sporobolus compositus* ), as well as forbs like *Lespedeza cuneata* , *Symphyotrichum ericoides* , and *Desmanthus illinoensis* (Shi et al. 2015; Castillioni et al. 2020). The northwestern sub‐site is lightly invaded by *B. ischaemum* and the southern sub‐site is more heavily invaded, but invasion levels are variable within the sub‐sites. We established 40 2 × 2 m observational plots in July 2019 that were divided between the two sub‐sites based on their size (plots 1–25 in the northwestern sub‐site and plots 26–40 in the southern sub‐site, Figure S1A,B). Plots were established along short transects (ranging from 11 to 23 m) of four to eight plots, with 1 m between adjacent plots and at least 5 m between transects. Plot‐level *B. ischaemum* cover ranged from 0% to 80%, with a mean of 23% cover.

### 2.3 Plot‐Level Plant Composition and Abundance

We recorded plant species composition (i.e., species identity and presence/absence) for each plot in July 2019. Species composition was recorded as binary presence/absence values for each species in each plot and species richness was recorded as the total number of species in each plot, excluding *B. ischaemum* . Abundance (percent foliar cover) was measured at the functional group level (C_3_ grasses, C_4_ grasses, herbaceous forbs, woody forbs, and legumes, as well as bare ground and litter) using cover class values adapted from Braun‐Blanquet (1932): Class 0 = 0% cover, Class 1 = 0%–1%, Class 2 = 1%–5%, Class 3 = 5%–25%, Class 4 = 25%–50%, Class 5 = 50%–75%, Class 6 = 75%–95%, Class 7 = 95%–100%. Since the interval between classes is unequal, we used the median value of each cover class in our analyses. Additionally, we calculated total foliar cover (the sum of all functional group cover values), subdominant foliar cover (the sum of all functional groups except C_4_ grasses), and a native C_4_ grass cover value (by subtracting the *B. ischaemum* percent‐cover value from the total C_4_ grass cover value) for each plot. Since the foliage of multiple functional groups often overlapped, the total coverage values of all functional groups in a plot often exceeded 100%. We recorded the cover of the invasive *B. ischaemum* at two levels of precision: a continuous estimate of *B. ischaemum* percent foliar cover for each plot (to take advantage of a regression approach) and a categorical assessment based on four broader cover classes which were used in compositional analyses. The four classes of *B. ischaemum* cover result from natural (i.e., unmanipulated) variation in the foliar cover of the invader across the invasion gradient. Fourteen plots had no *B. ischaemum* cover (Zero), while nine plots had Low (1% to 15% cover), nine plots had Medium (16% to 49% cover), and eight plots had High (50% cover and above) *B. ischaemum* abundance. Following this two‐tiered classification scheme, a plot that was observed to have 65% *B. ischaemum* cover, for example, would also be given a categorical value of “High.”

### 2.4 Assessing Within‐Species Impacts on *S. scoparium*

We assessed the impact of the invasive *B. ischaemum* on the abundance and functional traits of individuals of the native C_4_ grass *Schizachyrium scoparium* . The abundance of *S. scoparium* was recorded at the same two levels of precision as *B. ischaemum* abundance. Aboveground individual‐ and leaf‐level functional traits of *S. scoparium* were measured to determine how the magnitude of invasion impacted intra‐specific trait variation and composition. We randomly selected three individuals of *S. scoparium* from each plot and tagged them so that repeated measures could be made when necessary. For each of these individuals, we measured their height as the distance from the ground to the tallest portion of each plant and individual foliar cover as the percent of the plot covered by the foliage of each individual plant. Three mature leaves from each individual were collected to calculate specific leaf area (SLA) and leaf dry matter content (LDMC). Fresh mass of each leaf was determined to 0.01 g, scanned at 300 dpi using WINfolia software (Regent Inc., Canada) to determine area, and finally dried at 80°C for at least 48 h before the dry mass was recorded. SLA was calculated as leaf area/dry mass and is reported as cm^2^ of leaf area per gram of dry leaf matter. LDMC was calculated as leaf dry mass/fresh mass and represents the fraction of total leaf mass accounted for by dry material. We used the plot‐level average of each trait in our analyses and also calculated plot‐level variance for each trait. These traits were selected because they can all be considered “effect traits” (Funk et al. 2016) that can directly or indirectly influence neighboring species and the surrounding ecosystem. Indirect effects arise when several functional traits compound into a trait syndrome that alter species, community, and ecosystem‐level responses (Kichenin et al. 2013).

### 2.5 Statistical Analyses

Since plots were divided between two sub‐sites, we first ran an ANOVA in R 3.6.1 (R Foundation for Statistical Computing, Vienna, Austria, 2019) to determine if the sub‐sites differed in *B. ischaemum* cover. The sub‐sites were different (*p* < 0.001, F = 39.54), so we used mixed effects models in our analyses, with sub‐site as a random effect (Gelman and Hill 2006). Linear mixed effects models were performed using the function “lmer” in the package “lme4” (Bates et al. 2015) in R. In several cases, the variance accounted for by sub‐site was equal to zero, so linear fixed effects models were constructed with the function “lm” from the “stats” package in R. The mixed effects models were used to test the impact of invasion on the following response variables: the abundance of *S. scoparium*, the abundance of several functional groups (C_3_ grasses, herbaceous forbs, woody plants, bare ground, and litter), species richness, and the value of two *S. scoparium* functional traits (LDMC and individual cover). The fixed effects models were used to test the impact of invasion on the total abundance of C_4_ grasses; the abundance of native C_4_ grasses and legumes; the amount of total and subdominant functional group cover; the values of two *S. scoparium* functional traits (SLA and height); and the variance of all four *S. scoparium* functional traits. Because preliminary results suggested that the impact on *S. scoparium* and legume cover might be driven by the presence/absence of *B. ischaemum* , we employed additional analyses to ensure that impacts were observed between low and highly invaded plots, rather than non‐ and highly invaded plots. We used linear fixed effects models to examine the impact on the variables only in plots with *B. ischaemum* , and one‐way ANOVAs when there was no linear relationship within invaded plots. We also performed a one‐way ANOVA to determine if variance in *S. scoparium* functional trait means was impacted by the cover classes of *B. ischaemum*. Finally, we ran our linear models excluding plots where *Lespedeza cuneata* was present, another invasive plant, to remove any confounding effects of another invader (Table S1).

To determine how varying levels of *B. ischaemum* invasion (both invasion category and continuous abundance) impact the composition of the plant community, we performed PERMANOVAs (Anderson 2001; Anderson et al. 2006) in PRIMER (Plymouth Marine Laboratory, UK), running each with 999 permutations. We tested the effect of *B. ischaemum* cover category (Zero, Low, Medium, High) on species composition (recorded as presence/absence), functional group composition (abundance of C_4_ grasses, C_3_ grasses, forbs, woody plants, and legumes), and *S. scoparium* trait composition (height, cover, SLA, LDMC). The *S. scoparium* trait values were standardized (trait value/maximum of the trait) before analysis. For significant PERMANOVAs, we then performed pairwise permutation tests to determine which *B. ischaemum* cover categories differed from one another and ran SIMPER analyses to determine which species, functional groups, or functional traits drive the dissimilarity between cover categories. Since a significant PERMANOVA result can indicate compositional differences due to both shifts in location and shifts in dispersion (Anderson 2001), we used permutational analysis of multivariate dispersions (PERMDISPs) to assess which results may be due to differences in dispersion within cover categories. Given that the results from our permutation analyses using *B. ischaemum* invasion category and invasion abundance were similar, we present the results from the former analyses. We chose principle coordinate analyses (PCoA) rather than non‐metric multidimensional scaling (NMDS) to visualize our data (species, functional group, functional composition) to maximize linear correlation between dissimilarity distances rather than maximizing non‐linear correlation between dissimilarities.

## 3 Results

### 3.1 Impacts of Invasion on *S. scoparium*

**FIGURE 1:**
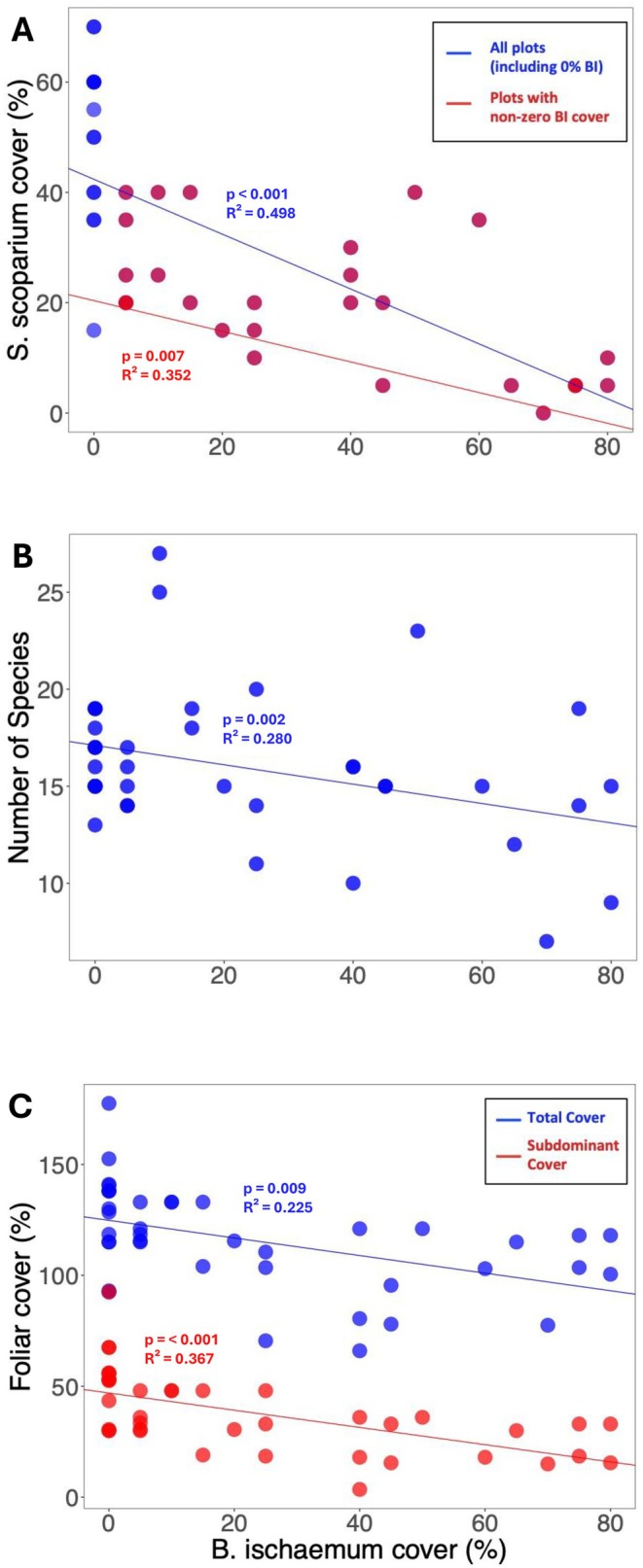
(A–C) Linear regression between increasing percent cover of *Bothriochloa ischaemum* and (A) percent cover of *Schizachyrium scoparium*, (B) species richness, and (C) percent foliar cover of the community and subdominant groups. (A) Data in red pertains to analyses excluding plots where *B. ischaemum* is absent; blue points pertain to plots with zero percent *B. ischaemum* cover; and the blue regression line includes all plots in the study. (B) Plots with zero or low *B. ischaemum* cover had an average of 16.43 ± 1.87 and 18.33 ± 4.69 species, respectively. Plots with medium or High *B. ischaemum* cover had an average of 14.67 ± 2.92 and 14.25 ± 5.15 species. (C) Data in blue pertains to analyses on total foliar cover, while data in red pertains to analyses on subdominant foliar cover. Total foliar cover can exceed 100% because the foliage of the different functional groups overlaps.

**FIGURE 2:**
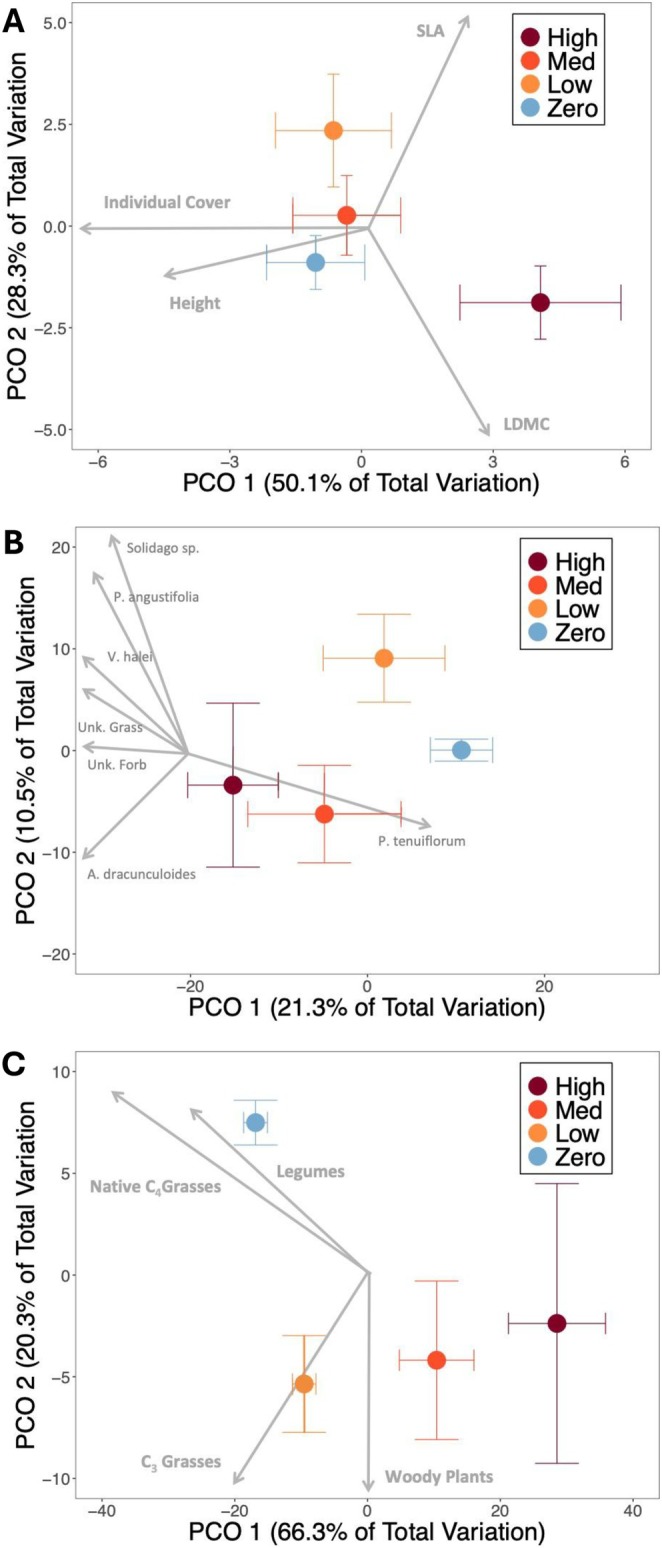
(A–C) Principal coordinate ordination for visualizing:(A) functional trait compositionof the native grass, *Schizachyrium scoparium*, (B) species composition, and (C) plant functional group composition of the native plant community across increasing foliar cover classes of the invasive grass *Bothriochloa* *ischaemum*. The cover classes are: Zero, low (1%–15%), medium (16%–49%), and high (50% or higher) cover. Points represent the centroids of each invasion category in the PCO axes and error bars represent the standard error. (A) The traits represented are height, individual foliar cover, specific leaf area (SLA), and leaf dry matter content (LDMC). PERMANOVA and SIMPER analyses indicate that trait composition in high invasion plots is significantly different from trait composition in zero and low invasion plots. (B) PERMANOVA and SIMPER analyses indicate that species composition in high invasion plots is significantly different from species composition in zero invasion plots. Vectors show species with a correlation of *r* > 0.7 to the axes: *Amphiachyris dranunculoides, Prunus angustifolia*, *Psoralidium teniflorum*, *Solidago* sp., *Verbena halei*, an unidentified grass, and an unidentified forb. Note that the axes explain only 31.8% of the variation in species composition, but the SIMPER analysis accounted for 100% of the variation. (C) PERMANOVA and SIMPER analyses indicate that all pairs of invasive cover class categories were significantly different from one another except for medium and high invasion plots. Vectors show functional groups with a correlation of *r* > 0.5 to the axes.

The invasive grass *Bothriochloa ischaemum* was negatively associated with the abundance of the native grass, *Schizachyrium scoparium* (Figure 1A, Table 1), with composition of its functional traits diverging with the highest abundance of the invasive grass relative to lower abundances (Figure 2A). As the foliar cover of the invasive grass increased, the foliar cover of native *S. scoparium* decreased linearly (*p* ≤ 0.001, *F* = 18.38). In the plots without the invasive species, the mean foliar cover of the native grass was 50% (range = 15%–70%), while in plots where the invader was present, the mean foliar cover of *S. scoparium* was only 20% (range = 0%–40%). There was a 59% reduction in mean foliar cover of *S. scoparium* from the uninvaded to the invaded plots. While it appeared that this effect might be driven simply by the presence/absence of *B. ischaemum* , an additional linear model focused only on plots where *B. ischaemum* was present still indicated a linear reduction in *S. scoparium* cover as *B. ischaemum* cover increases (*p* = 0.007, *R* ^2^ = 0.352, Figure 1A, Table 1).

**TABLE 1:** Parameters from all analyses run for this project.

Linear fixed effects models	*p*	*R*‐squared	Intercept	Slope	*F*
SS cover ~ non‐zero BI cover	0.007	0.352	31.03	−0.244	6.24
Mean SS height	0.093	0.124	46.81	−0.067	2.54
Mean SS SLA	0.675	0.022	169.61	−0.029	0.40
Total C4 grass median cover	0.664	0.219	78.22	0.057	0.41
Native C4 grass median cover	< 0.001	0.844	78.22	−0.943	100.30
Legume median cover	< 0.001	0.331	24.67	−0.247	9.13
Legume median cover ~ non‐zero BI cover	0.460	0.653	10.81	−0.066	0.80
Total foliar cover	0.009	0.225	124.89	−0.386	5.38
Subdominant foliar cover	< 0.001	0.367	46.67	−0.443	10.72
SS cover	< 0.001	0.498	42.96	−0.400	18.38
Mean SS individual cover	0.130	0.107	1.95	−0.009	2.16
Mean SS LDMC	0.426	0.046	0.44	< 0.001	0.87
Species richness	0.002	0.280	16.76	−0.105	7.19
C3 grass median cover	0.055	0.146	3.77	−0.096	3.15
Herbaceous forb median cover	0.261	0.070	15.44	−0.002	1.39
Woody plant median cover	0.010	0.219	2.79	−0.099	5.18
Bare ground	0.524	0.035	10.03	−0.082	0.66
Litter	0.340	0.058	22.64	0.161	1.11

*Note:* All linear models were run with the percent foliar cover of the invasive grass *Bothriochloa ischaemum* as the independent variable and within‐species (population level) or across‐species (community‐level) metrics as responses. The four *B. ischaemum* cover categories (Zero, Low (1%–15% cover), Medium (16%–49% cover), and High (50% or higher cover)) were used for the PERMANOVAs and PERMDISPs. Abbreviations: BI = *Bothriochloa ischaemum* , LDMC = leaf dry matter content, SLA = specific leaf area, SS = *Schizachyrium scoparium* .

Increased *B. ischaemum* cover was also negatively associated with the average height of *S. scoparium* and greater dissimilarity in functional trait composition. The average height of *S. scoparium* decreased (*p* = 0.093, *R* ^2^ = 0.124, Table 1, Figure S2A) as *B. ischaemum* cover increased. However, the mean of the other functional traits did not change significantly across the invasion gradient: individual foliar cover (*p* = 0.13), SLA (*p* = 0.675), LDMC (*p* = 0.426, Figure S2B,C). Increasing *B. ischaemum* cover was significantly associated with a shift in functional trait composition of *S. scoparium* individuals (*p* = 0.031, Figure 2A). Pairwise tests showed that plots with High *B. ischaemum* cover (above 50% foliar cover) were significantly different from plots with Zero *B. ischaemum* (*p* = 0.031) and Low (1%–15%) *B. ischaemum* cover (*p* = 0.014). Plots in the Medium category did not differ from any other category. The individual height and foliar cover accounted for 67% of the dissimilarity between Zero and High *B. ischaemum* cover, while individual foliar cover and SLA accounted for 71% of the dissimilarity between Low and High *B. ischaemum* cover. The one‐way ANOVAs on trait variance confirmed that there was no impact of invasion category on trait variance (all *p*‐values above 0.15). The main differences when *Lespedeza* was excluded were strengthening effects of the invasion gradient on native population height and weakening effects of the invasion gradient on community level woody cover. We then proceeded with the linear models that included all plots across our subsites (Table S1).

### 3.2 Impacts of Invasion on the Native Plant Community

Invasion by *B. ischaemum* was negatively associated with several diversity characteristics of the native plant community and altered the compositional similarity of both species and functional groups. As *B. ischaemum* foliar cover increased, the total species richness declined (*p* < 0.001, *F* = 13.26, Figure 1B). Plots with Zero or Low *B. ischaemum* cover had an average of 16.43 ± 1.87 and 18.33 ± 4.69 species, respectively. Plots with Medium or High *B. ischaemum* cover had an average of 14.67 ± 2.92 and 14.25 ± 5.15 species. There was also a significant effect of *B. ischaemum* cover on the species composition (Figure 2B) of these plots (*p* = 0.013). Pairwise tests indicated that only the plots in the Zero and High categories of *B. ischaemum* cover differed significantly (*p* = 0.0003). Table 2 indicates the species that contribute the most to the dissimilarity between these two categories. Legumes such as *Dalea purpurea* and *Psoralidium tenuiflorum* , and C_3_ grasses such as *Dichanthelium oligosanthes,* were found more frequently in plots with zero *B. ischaemum* cover compared to plots with high *B. ischaemum* . Conversely, species such as *Chamaecrista fasciculata* (legume) and *Croton monanthogynus* (forb) were found more often in plots with High *B. ischaemum* compared to plots with Zero cover. Finally, the PERMDISP results indicated that there was greater within‐cover‐class dispersion for plots with Medium invasion (mean = 44.4) compared to zero (mean = 34.1; *p* = 0.004) and low (mean = 39.7; *p* = 0.025) invasion and greater within‐cover‐class dispersion for plots with high invasion (mean = 46.1) compared to zero invasion (*p* = 0.015).

**TABLE 2:** Results from SIMPER analysis addressing species‐specific contributions to the overall dissimilarity in species composition between uninvaded (Zero) and highly invaded (High) plots.

Species (functional group)	Frequency (zero invasion)	Frequency (high invasion)	Percent contribution	Cumulative dissimilarity
*Dalea purpurea* (legume)	0.93	0.13	4.87	4.87
*Psoralidium tenuiflorum* (legume)	1.00	0.38	3.64	8.51
*Dichanthelium oligosanthes* (C_3_ grass)	0.57	0.13	3.34	11.84
Unknown Forb (forb)	0.43	0.75	3.20	15.04
*Liatris squarrosa* (forb)	0.93	0.50	3.17	18.21
*Chamaecrista fasciculata* (legume)	0.21	0.63	3.11	21.31
*Stenaria nigricans* (forb)	0.71	0.50	3.04	24.35
Unknown Grass	0.36	0.63	3.03	27.38
*Erigeron strigosus* (forb)	0.86	0.50	3.02	30.40
*Croton monanthogynus* (forb)	0.43	0.50	2.90	33.30
*Tragia betonicifolia* (forb)	0.43	0.50	2.88	36.18
*Oenothera serrulata* (forb)	0.43	0.38	2.78	38.97
*Solidago rigida* (forb)	0.36	0.38	2.77	41.73
*Desmanthus illinoensis* (legume)	0.64	0.63	2.71	44.44
*Sorghastrum nutans* (C_4_ grass)	0.93	0.63	2.57	47.01
*Amphiachyris dracunculoides* (forb)	0.00	0.38	2.51	49.52
*Solidago nemoralis* (forb)	0.93	0.63	2.46	51.98

*Note:* The species contributing 50% of the total dissimilarity in the species composition between Zero and High invasion plots are shown. No other pairs of cover categories differed significantly in their species composition. “Frequency” gives the proportion of plots in each cover category in which the species was present; “% Contribution” gives the percent of the dissimilarity between Zero and High invasion plots accounted for by the frequency of each species; “Cumulative Dissimilarity” gives the percent of dissimilarity between Zero and High invasion plots accounted for by each species and all species listed above. Note that species that are abundant at all invasion levels (such as *Ambrosia psilostachya* ) are not major contributors to dissimilarity.

As *B. ischaemum* cover increased, several plant functional groups declined in abundance (Figure 2C, Tables 1 and S1). Unsurprisingly, the cover of native C_4_ grasses dramatically declined as *B. ischaemum* cover increased. About 84% of the total variation in native C_4_ grass cover was explained by the invader increase (*p* < 0.001, *R* ^2^ = 0.84, Table 1). The slope of this relationship was −1.01, indicating a 1:1 replacement of native C_4_ grasses with the invasive C_4_ grass. There was also a significant decline in C_3_ grass cover, although this effect was relatively weak (*p* = 0.039, F = 5.26, Table 1). There appeared to be a decline in legume foliar cover as *B. ischaemum* cover increased (*p* < 0.001, *R* ^2^ = 0.32, Table 1), but this relationship did not hold up when we only looked at invaded plots (*p* = 0.209). A one‐way ANOVA showed there was a difference in legume cover based on *B. ischaemum* invasion category, and a Tukey HSD test in R indicated that plots with zero invasion differed from all invasion levels (all *p*‐values < 0.001), but none of the invasion categories differed from one another (all *p*‐values > 0.74). Legumes were subdominant in all plots but accounted for a substantially greater proportion of total foliar cover in uninvaded plots compared to invaded plots. Legumes had a mean foliar cover of 33% (range = 15%–63%) in uninvaded plots but only 9% (range = 0%–15%) in plots with *B. ischaemum* , a 74% reduction in cover. The other functional groups (total C_4_ grass cover, herbaceous forbs, and woody plants), as well as the amount of bare ground and litter in each plot, did not vary in cover across the invasion gradient (all *p*‐values > 0.062). However, increasing invasion by *B. ischaemum* reduced the total foliar cover of each plot (*p* = 0.002, *R* ^2^ = 0.23, Figure 1C) and the total foliar cover of subdominants (all functional groups except C_4_ grasses; *p* < 0.001, *R* ^2^ = 0.36, Figure 1C). This reduction in total foliar cover did not lead to an increase in bare ground because the foliage of the different plants overlapped and led total foliar cover to often exceed 100%.

**FIGURE 3:**
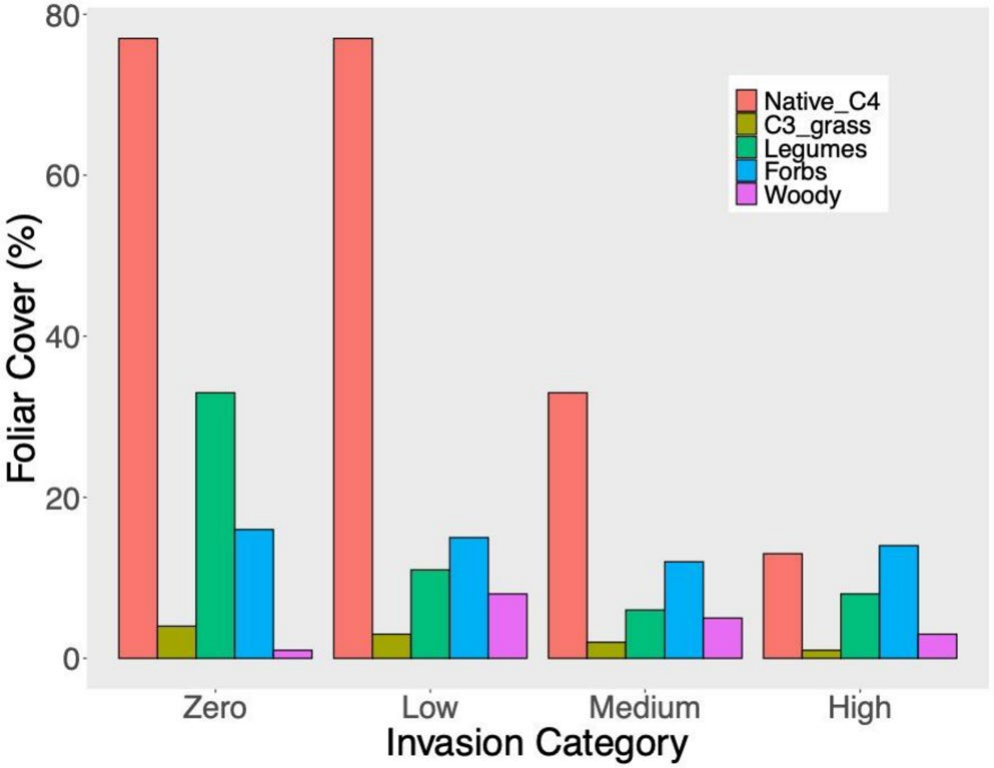
Functional group cover varied with cover category of the invasive grass *Bothriochloa ischaemum*. The cover of C3 grasses, herbaceous forbs, and woody plants was unaffected by invasion. Native C4 grass cover declined linearly as invasion increased (*p* < 0.001, *R* ^2^ = 0.84), while the decline in legume cover was driven by the difference between invaded and uninvaded plots. A one‐way ANOVA showed there was a difference in legume cover based on *B. ischaemum* invasion category, and a Tukey HSD test indicated that plots with Zero invasion differed from all invasion levels (all *p*‐value < 0.001).

We found significant variation in the relative abundance of plant functional groups across *B. ischaemum* cover categories (*p* = 0.0001). Pairwise tests indicated that all categories of *B. ischaemum* cover differed from one another except for Medium and High invasion plots (Figure 3). Plots with Zero *B. ischaemum* differed from all other levels of *B. ischaemum* cover (Low, *p* = 0.0007; Medium, *p* = 0.0001; High, p = 0.0001). Plots with Low *B. ischaemum* cover also differed from plots with Medium (*p* = 0.0054) and High (p = 0.0001) cover of the invasive species. Decreases in native C_4_ grass and legume cover as invasion increased accounted for most of the dissimilarity between invasion categories.

There were also significant differences in the dissimilarity of functional group abundance for plots *within* the different invasion cover classes. There was significantly more dispersion within the Medium (mean = 20.1) and High (mean = 27.2) invasion plots than in the Zero (mean = 10.6) or Low (mean = 8.1) invasion plots. Dispersion within plots with Zero invasion differed from the dispersion within plots with Medium (*p* = 0.002) and High (*p* = 0.002) invasion. Plots with Low invasion also differed from plots with Medium (*p* = 0.002) and High (*p* = 0.001) invasion, indicating that functional group composition is more consistent within plots with Zero‐Low *B. ischaemum* invasion and more variable within plots with Medium‐High invasion.

## 4 Discussion

Invasive species are a well‐known threat to diversity and function *across* a variety of ecosystems (Mack et al. 2000; Ricciardi et al. 2013), but proper assessment of their ecological impacts *within* individual systems on a trait‐ and species‐level basis is key to developing individualized management strategies (Drenovsky et al. 2012). Our study shows that the invasive C_4_ grass *Bothriochloa ischaemum* has significant negative associations in within‐species (on a population of native *Schizachyrium scoparium* ) and across‐species (on community metrics of a mixed‐grass prairie) properties with potential negative impacts across levels of organization. Increasing invasion−i.e. abundance−by *B. ischaemum* was associated with significant reduction in both the abundance and height of the native *S. scoparium* . Individuals of *S. scoparium* growing in high‐invasion plots had a dissimilar suite of functional trait values from individuals growing in uninvaded plots, with decreasing trait values leading toward smaller plants with less acquisitive traits in highly invaded plots. Further, *B. ischaemum* invasion was related to reduced species richness of the native plant community and caused declines in native C_4_ grass cover, C_3_ grass cover, legume cover, total foliar cover, and subdominant species cover. Invasion was correlated wi†h significant differences in the species composition of uninvaded and highly invaded plots, as well as dissimilarity in functional group composition across most levels of invasion. In general, the *within‐species* impacts were stronger than *across‐species* impacts, which has interesting implications for how impact scales across levels of organization. Taken together, population‐level impacts due to plant invasions may be detected before community‐level impacts (Vilà et al. 2011).

The finding that increasing *B. ischaemum* is associated with reduced size and abundance of *S. scoparium* supports our prediction of a negative relationship between exotic and native grass abundance and confirms the harmful impacts of this invader on co‐occurring dominant native grasses (Schmidt et al. 2008; Robertson and Hickman 2012; Duell et al. 2016). Limiting similarity, given functional group overlap of invader (*Botriochloa*) and native dominant species (*Schizachyrium*) may have contributed to the negative association as shown in other invasion studies (Emery 2007). Alternatively, *Bothriochloa ischaemum* invasion can indirectly inhibit the growth of native dominant ( *S. scoparium* ) as well as other native warm‐season C4 grasses by altering the arbuscular mycorrhiza fungal community on which they depend (Duell et al. 2016), an effect that can continue even after the invader has been removed (Wilson et al. 2012).

We also found support for our prediction that invasion would lead to shifts in trait composition of *S. scoparium* . Our study is the first to show that high invasion by *B. ischaemum* alters the functional trait composition of a native species, building on previous work that showed invasion could alter trait composition at the community level (Spyreas et al. 2010; Sodhi et al. 2019). In assessing the effects of functional traits on ecosystem processes, it is important to consider changes in trait syndromes rather than just single traits (Kichenin et al. 2013). While the mean of three of the four traits we measured (SLA, LDMC, and individual cover) was not significantly impacted when analyzed individually, the shifts in these traits compounded one another, resulting in a dissimilar suite of traits in high‐invasion patches compared to uninvaded patches. The *S. scoparium* individuals that persist under High invasion are smaller (lower height and individual cover) and exhibit more signs of resource stress (lower SLA and higher LDMC; Cornelissen et al. 2003) than individuals growing under Zero or Low invasion. Additionally, although the impacts on height and trait composition are relatively weak, they are likely amplified by the simultaneous strong impact on abundance, potentially causing serious consequences for *S. scoparium* growing in invaded systems. Inherent functional trait variation in dominant species can also result from environmental gradients prior to invader arrival and/or establishment (Ren et al. 2024).

Plant height is correlated with aboveground biomass, fecundity, and competitive vigor and can provide an estimate of reproductive fitness (Younginger et al. 2017). This relationship could lead to a positive feedback loop that promotes further invasion of *B. ischaemum* if *S. scoparium* individuals growing in heavily invaded habitats have lower fitness. This cycle could be exacerbated by the ability of *B. ischaemum* to directly and indirectly inhibit *S. scoparium* growth through potential allelopathic compounds. Leachate from *B. ischaemum* significantly reduced germination of *S. scoparium* seeds by 91%, as well as the survival and biomass accumulation of *S. scoparium* seedlings (Greer et al. 2014). The replacement of the native *S. scoparium* with the exotic *B. ischaemum* is likely to have negative impacts on the broader community and ecosystem as well. The direct ecological influences of these two species are poorly studied, but some general predictions can be made. *Bothriochloa ischaemum* has lower‐quality litter and a shorter, but more productive, growing season than *S. scoparium* , which can lower plant‐available soil nitrogen and increase aboveground net primary productivity of invaded sites (Basham 2013). The decreased species richness and lower abundance of subdominant C_4_ grasses and legumes that we observed suggests that *B. ischaemum* creates a less favorable microhabitat for subdominant native plant species, reducing the available nitrogen and light by increasing canopy cover relative to *S. scoparium* . Our results showed that *B. ischaemum* invasion reduces plant species richness, the abundance of native C_3_ and C_4_ grasses, the abundance of legumes, and both total foliar cover and subdominant foliar cover, but that invasion does not impact herbaceous forb or woody plant cover, suggesting that the impact of *B. ischaemum* invasion on the community is variable but generally negative. Interestingly, although total C_4_ grass cover remained constant across all plots, areas dominated by the *invasive* C_4_ grass had significantly lower species richness, total cover, and subdominant cover than areas with the equivalent abundance of the *native* C_4_ grass. These observations suggest that a 1:1 replacement of native grasses with exotic grasses does not always correspond to an equivalent role in the community.

Invasion not only reduced the abundance of native species but also led to shifts in species and functional group composition of the community. Fargione et al. (2003) showed that native species were most successful at hindering invasion by exotic species from the same functional group, and that native C_4_ grasses are the functional group that has the strongest negative impact on exotic species from other functional groups. Stuble and Young (2020) show that native grasses have stronger priority effects than native forbs during restoration to reduce invasion in the long run. Our results suggest that if the invader is a strong enough competitor, the opposite is true as well: *B. ischaemum* has the strongest negative impact within its functional group (native C_4_ grasses, similar to Damasceno et al. 2018) but also exerts negative impacts on other functional groups such as legumes. Although the reductions in C_3_ grass cover were relatively weak, they combined with reductions in native C_4_ grass and legume cover to produce significantly different species and functional group compositions. These changes in composition can have significant impacts on nitrogen cycling (Mack et al. 2001). The loss of legumes could be a particularly detrimental effect of *B. ischaemum* invasion, potentially altering the nutrient cycling of invaded areas that can no longer support these nitrogen fixers (Knops et al. 2002). Nitrogen accumulation rates are positively related to legume cover but negatively related to C_3_ grass and forb cover (Knops and Tilman 2000), so the loss of legumes is likely to compound the decrease in available nitrogen caused directly by *B. ischaemum* (Basham 2013). Another intriguing result of our study is that there is greater dispersion within the Medium and High invasion plots compared to Zero and Low invasion plots, indicating greater heterogeneity of species and functional group composition at increased levels of invasion. In other words, the composition of all uninvaded plots was essentially the same, but highly invaded plots differed greatly from one another. Although plant invasions have decreased variation *across* sites (McKinney 2004; Baiser et al. 2012; Sodhi et al. 2019), our results suggest that they can increase variation *within* individual sites. Due to the nature of our study as a natural experiment taking advantage of natural variation in exotic species abundance, our results, taken in isolation, cannot conclusively state that invasive species abundance caused the observed differences in community and intraspecific composition (rather than differences in the community causing variations in invader abundance). However, in light of previous field studies that confirm the patterns of impact from *B. ischaemum* (Gabbard and Fowler 2007; Robertson and Hickman 2012) and greenhouse studies that provide mechanisms for impact (Schmidt et al. 2008; Greer et al. 2014), it is ecologically justifiable to conclude that the observed impacts are caused by *B. ischaemum* invasion.

There have been repeated calls for invasion impact studies to assess impacts over a gradient of invader abundance (Ehrenfeld 2010; Vilà et al. 2011; Hulme et al. 2013; Scasta et al. 2015) and our study contributes to filling this knowledge gap by demonstrating that the role of invader abundance varies depending on the impact of interest. The majority of the impacts we found (on *S. scoparium* abundance and height, species richness, native C_3_ and C_4_ grass cover, total cover, and subdominant cover) increase linearly as the abundance of the invader increases. The impact on legume abundance, however, was not related to invader abundance. Rather, the threshold for impact is very low (1%–15% cover of the invader), but the magnitude of this impact is constant across the abundance gradient. This pattern has important implications for the management of *B. ischaemum* . If the abundance of this invader can be kept low, its impacts may be negligible for most native species even if it cannot be completely eradicated. However, complete eradication of the invader may be the only way to protect some native species. Furthermore, our results expand on the pattern observed by Vilà et al. (2011). They found that impacts on communities are more severe than impacts on ecosystem processes, which suggests that by the time ecosystem impacts are detected, communities have likely already sustained severe impacts. In this study, impacts on the native community, such as declines in species richness and changes in species or functional group composition, are less severe (in terms of *F*‐value) than the impact on the native dominant species, *S. scoparium* , suggesting a progression of impact severity from species > community > ecosystem. This pattern could have important implications for invasive species management and monitoring if impacts on communities and ecosystems are consistently preceded by strong impacts on the dominant native plants.

## 5 Conclusions

Biological invasions and their associated ecological impacts will continue to be a pressing challenge to biodiversity conservation. We contribute to addressing this challenge by demonstrating the importance of several characteristics of invasion impacts. First, as the abundance of an invasive grass increased, the abundance of a dominant native grass (along with the other co‐occurring native grasses) linearly declined. Secondly, increasing the abundance of an invasive grass altered the functional traits of dominant native plants, with native plant function shifting toward resource conservation rather than resource acquisition. Understanding the implications of these results can help guide informed management strategies. Future studies should track functional traits at the community level, in conjunction with ecosystem variables, along invasion gradients to determine ecological impacts across levels of organization.

## Author Contributions

**Joshua D. Kouri:** conceptualization (lead), data curation (lead), formal analysis (lead), funding acquisition (lead), investigation (lead), methodology (lead), project administration (lead), software (lead), supervision (lead), validation (lead), visualization (lead), writing – original draft (lead), writing – review and editing (lead). **Emma Rust:** validation (supporting), visualization (supporting), writing – review and editing (supporting). **Lara Souza:** conceptualization (equal), data curation (equal), formal analysis (equal), funding acquisition (equal), investigation (equal), methodology (equal), project administration (equal), resources (lead), software (supporting), supervision (supporting), validation (supporting), visualization (supporting), writing – original draft (equal), writing – review and editing (equal).

## Funding

The work was partially supported by grants from NSF EArly‐concept Grants for Exploratory Research (EAGER) (NSF Award #1745404), the University of Oklahoma Graduate Student Senate, and the University of Oklahoma Biological Station Graduate Summer Research Fellowship.

## Conflicts of Interest

The authors declare no conflicts of interest.

## Supporting information


**Figure S1:** Map of plot locations at sub‐site 1 (A) and 2 (B) at the Kessler atmospheric and ecological field station. Plots are arranged in short transects (10–15 m) of four to five plots. Each plot is 2 × 2 m. Twenty‐five plots are located at sub‐site 1 and 15 in subsite 2. Plot color indicates invasion level: blue = Zero invasion, yellow = Low (1%–15%) invasion, orange = Medium (16%–49%) invasion, and red = High (50% and over) invasion. Subsite 1 centered at N34.9816 W‐97.5323 and subsite 2 at N34.9755 W‐97.5222.

**Figure S2:** (A–C) Linear regression between increasing percent cover of *Bothriochloa ischaemum* and (A) mean height of *Schizachyrium scoparium* , (B) mean specific leaf area (SLA) of *S. scoparium* , and (C) mean leaf dry matter content (LDMC) of *S. scoparium* . All *p*‐values are above 0.05 and represented by dashed lines.

**Table S1:** Parameters from all analyses run for this project. All linear models were run with the percent foliar cover of the invasive grass *Bothriochloa ischaemum* as the independent variable and within‐species (population level) or across‐species (community‐level) metrics as responses excluding two plots that had *Lespedeza cuneata* 's presence. The four *B. ischaemum* cover categories (Zero, Low (1%–15% cover), Medium (16%–49% cover), and High (50% or higher cover)) were used for the PERMANOVAs and PERMDISPs. “SS” = *Schizachyrium scoparium* ; “BI” = *Bothriochloa ischaemum* ; “SLA” = specific leaf area; “LDMC” = leaf dry matter content.

## Data Availability

The datasets generated during and/or analyzed during the current study are available https://doi.org/10.5281/zenodo.17537736.
