# Supplementary material for: Intra‐ and Inter‐Specific Ecological Impacts Vary Across a Gradient of Abundance of an Invasive Species, Bothriochloa ischaemum, in a Mixed‐Grass Prairie
Source: Ecol Evol. 2026 Mar 10;16(3):e73212. doi: 10.1002/ece3.73212 (PMC12975291; doi:10.1002/ece3.73212)

Fig. S1 Map of plot locations at sub-site 1 (A) and 2 (B) at the Kessler Atmospheric and Ecological Field Station. Plots are arranged in short transects (10 – 15m) of four to five plots. Each plot is 2m x 2m. Twenty-five plots are located at sub-site 1 and 15 in subsite 2. Plot color indicates invasion level: blue = Zero invasion, yellow = Low (1 – 15%) invasion, orange = Medium (16 – 49%) invasion, and red = High (50% and over) invasion. Subsite 1 centered at N34.9816 W-97.5323 and subsite 2 at N34.9755 W-97.5222.

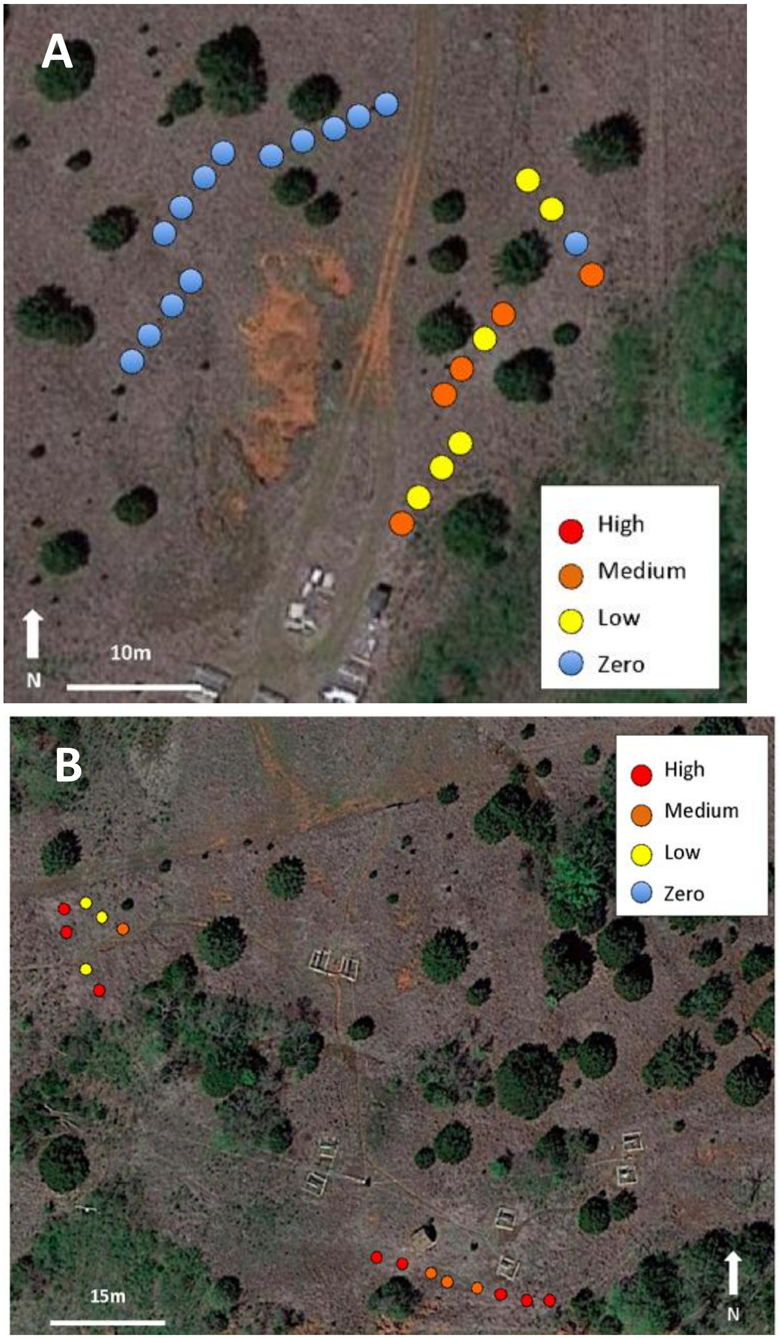

Supplement: Supplementary file 1 — Figure S1: Map of plot locations at sub‐site 1 (A) and 2 (B) at the Kessler atmospheric and ecological field station. Plots are arranged in short transects (10–15 m) of four to five plots. Each plot is 2 × 2 m. Twenty‐five plots are located at sub‐site 1 and 15 in subsite 2. Plot color indicates invasion level: blue = Zero invasion, yellow = Low (1%–15%) invasion, orange = Medium (16%–49%) invasion, and red = High (50% and over) invasion. Subsite 1 centered at N34.9816 W‐97.5323 and subsite 2 at N34.9755 W‐97.5222. [file ECE3-16-e73212-s001.pdf]
