# Supplementary material for: Intra‐ and Inter‐Specific Ecological Impacts Vary Across a Gradient of Abundance of an Invasive Species, Bothriochloa ischaemum, in a Mixed‐Grass Prairie
Source: Ecol Evol. 2026 Mar 10;16(3):e73212. doi: 10.1002/ece3.73212 (PMC12975291; doi:10.1002/ece3.73212)
Supplement: Supplementary file 2 — Figure S2: (A–C) Linear regression between increasing percent cover of Bothriochloa ischaemum and (A) mean height of Schizachyrium scoparium , (B) mean specific leaf area (SLA) of S. scoparium , and (C) mean leaf dry matter content (LDMC) of S. scoparium . All p‐values are above 0.05 and represented by dashed lines. [file ECE3-16-e73212-s002.pdf]

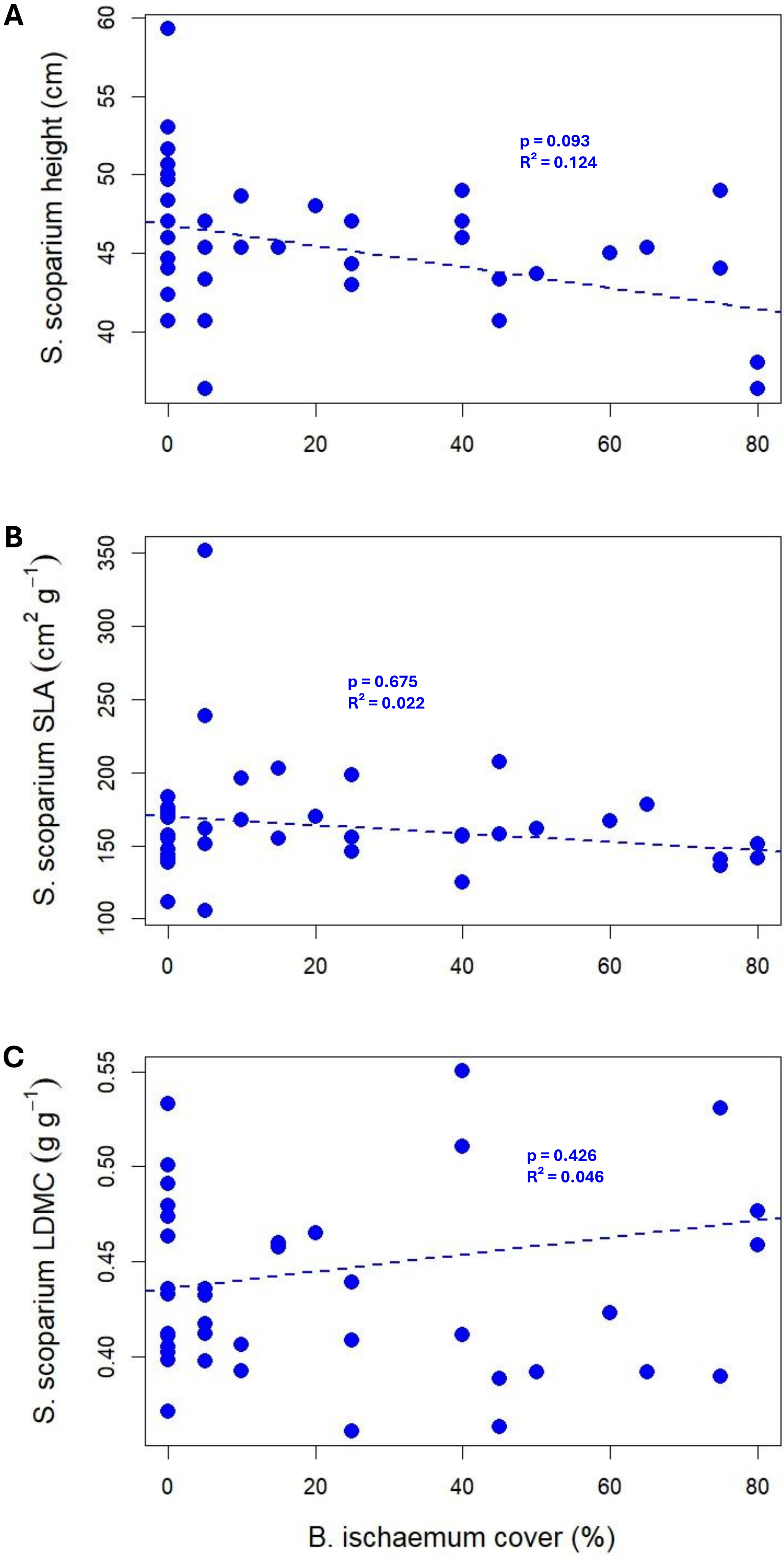

Fig. S2A-C Linear regression between increasing percent cover of *Bothriochloa ischaemum* and A) mean height of *Schizachyrium scoparium*, B) mean specific leaf area (SLA) of *S. scoparium*, and C) mean leaf dry matter content (LDMC) of *S. scoparium*. All p-values are above 0.05 and represented by dashed lines.
