# Supplementary material for: Intra‐ and Inter‐Specific Ecological Impacts Vary Across a Gradient of Abundance of an Invasive Species, Bothriochloa ischaemum, in a Mixed‐Grass Prairie
Source: Ecol Evol. 2026 Mar 10;16(3):e73212. doi: 10.1002/ece3.73212 (PMC12975291; doi:10.1002/ece3.73212)
Supplement: Supplementary file 3 — Table S1: Parameters from all analyses run for this project. All linear models were run with the percent foliar cover of the invasive grass Bothriochloa ischaemum as the independent variable and within‐species (population level) or across‐species (community‐level) metrics as responses excluding two plots that had Lespedeza cuneata 's presence. The four B. ischaemum cover categories (Zero, Low (1%–15% cover), Medium (16%–49% cover), and High (50% or higher cover)) were used for the PERMANOVAs and PERMDISPs. “SS” = Schizachyrium scoparium ; “BI” = Bothriochloa ischaemum ; “SLA” = specific leaf area; “LDMC” = leaf dry matter content. [file ECE3-16-e73212-s003.pdf]

Table S1. Parameters from all analyses run for this project. All linear models were run with the percent foliar cover of the invasive grass *Bothriochloa ischaemum* as the independent variable and within-species (population level) or across-species (community-level) metrics as responses excluding two plots that had *Lespedeza cuneata's* presence. The four *B. ischaemum* cover categories (Zero, Low (1–15% cover), Medium (16–49% cover), and High (50% or higher cover)) were used for the PERMANOVAs and PERMDISPs. "SS" = *Schizachyrium scoparium*; "BI" = *Bothriochloa ischaemum*; "SLA" = specific leaf area; "LDMC" = leaf dry matter content.

Linear Fixed Effects Models (Excluding *Lespedeza cuneata*)	**p**	**R-squared**	**Intercept**	**Slope**	**F**
SS cover ~ non-zero BI cover	0.009	0.348	31.05	-0.246	5.871
mean SS height	0.059	0.153	47.14	-0.077	3.072
mean SS SLA	0.708	0.020	169.11	-0.294	0.349
total C4 grass median cover	0.497	0.039	77.58	0.098	0.714
native C4 grass median cover	<0.001	0.844	77.58	-0.902	94.490
legume median cover	0.001	0.317	24.13	-0.246	8.131
legume median cover ~ non-zero BI cover	0.557	0.052	10.47	-0.049	0.600
total foliar cover	0.017	0.207	123.21	-0.326	4.576
subdominant foliar cover	<0.001	0.351	45.63	-0.424	9.444
SS cover	<0.001	0.548	44.47	-0.439	21.200
mean SS individual cover	0.078	0.140	2.00	-0.010	2.756
mean SS LDMC	0.595	0.030	0.44	<0.001	0.528
species richness	0.017	0.207	16.52	-0.088	4.575
C3 grass median cover	0.045	0.163	3.32	-0.099	3.395
herbaceous forb median cover	0.253	0.076	15.39	0.005	1.429
woody plant median cover	0.046	0.162	2.79	-0.085	3.379
bare ground	0.240	0.080	10.87	-0.132	1.488
litter	0.382	0.055	21.97	0.168	0.989
